# Evaluation of the Canadian Clinical Practice Guidelines Risk Prediction Tool for Acute Aortic Syndrome: The RIPP Score

**DOI:** 10.1155/2023/6636800

**Published:** 2023-05-25

**Authors:** Robert Ohle, Sarah McIsaac, Madison Van Drusen, Aaron Regis, Owen Montpellier, Mackenzie Ludgate, Oluwadamilola Bodunde, David W. Savage, Krishan Yadav

**Affiliations:** ^1^The Department of Emergency Medicine, Health Science North Research Institute, Northern Ontario School of Medicine University, Sudbury, Ontario, Canada; ^2^Department of Critical Care, Department of Anaesthesia, Northern Ontario School of Medicine University, Sudbury, Ontario, Canada; ^3^Department of Undergraduate Medicine, University of Ottawa, Ottawa, Ontario, Canada; ^4^Department of Undergraduate Medicine, Northern Ontario School of Medicine, Sudbury, Ontario, Canada; ^5^Clinical Sciences Division, Nortner Ontario School of Medicine University, Thunder Bay, Ontario, Canada; ^6^Clinical Epidemiology Program, Ottawa Hospital Research Institute, Ottawa, Ontario, Canada

## Abstract

**Introduction:**

Acute aortic syndrome (AAS) is a rare clinical syndrome with a high mortality rate. The Canadian clinical practice guideline for the diagnosis of AAS was developed in order to reduce the frequency of misdiagnoses. As part of the guideline, a clinical decision aid was developed to facilitate clinician decision-making (RIPP score). The aim of this study is to validate the diagnostic accuracy of this tool and assess its performance in comparison to other risk prediction tools that have been developed.

**Methods:**

This was a historical case-control study. Consecutive cases and controls were recruited from three academic emergency departments from 2002–2020. Cases were identified through an admission, discharge, or death certificated diagnosis of acute aortic syndrome. Controls were identified through presenting complaint of chest, abdominal, flank, back pain, and/or perfusion deficit. We compared the clinical decision tools' C statistic and used the DeLong method to test for the significance of these differences and report sensitivity and specificity with 95% confidence intervals.

**Results:**

We collected data on 379 cases of acute aortic syndrome and 1340 potential eligible controls; 379 patients were randomly selected from the final population. The RIPP score had a sensitivity of 99.7% (98.54–99.99). This higher sensitivity resulted in a lower specificity (53%) compared to the other clinical decision aids (63–86%). The DeLong comparison of the C statistics found that the RIPP score had a higher C statistic than the ADDRS (−0.0423 (95% confidence interval −0.07–0.02); *P* < 0.0009) and the AORTAs score (−0.05 (−0.07 to −0.02); *P* = 0.0002), no difference compared to the Lovy decision tool (0.02 (95% CI −0.01–0.05 *P* < 0.25)) and decreased compared to the Von Kodolitsch decision tool (0.04 (95% CI 0.01–0.07 *P* < 0.008)).

**Conclusion:**

The Canadian clinical practice guideline's AAS clinical decision aid is a highly sensitive tool that uses readily available clinical information. It has the potential to improve diagnosis of AAS in the emergency department.

## 1. Introduction

Acute aortic syndrome is a time-sensitive emergency defined by three distinct diagnoses: aortic dissection, intramural hematoma, and penetrating atherosclerotic ulcer. Acute aortic syndrome and each of its component diagnoses involve blood leaking into the wall of the aorta, which is the major artery that supplies blood to the entire body [1]. This blood separates the layers of the wall of the aorta and can block blood flow to vital organs (heart, muscle, brain, or limbs) or lead to a rupture of the aorta and catastrophic blood loss [2–5]. The risk of death from acute aortic syndrome increases by 2% per hour, reaching 90% if undiagnosed [6]. Currently, there is significant variation in how physicians investigate acute aortic syndrome. This variation has led to inefficient use of computed tomographic imaging and a high miss rate. Health care providers miss 1 in 4 cases on the first presentation [2, 7–9]. Computed tomography is the investigation of choice to diagnose acute aortic syndrome. Its use in the emergency department has increased exponentially over the past number of years without any impact on the number of missed cases [10].

We have developed the Canadian clinical practice guideline for the diagnosis of acute aortic syndrome in the emergency department [11]. The goal of this guideline is to offer practical recommendations to guide the investigation of acute aortic syndrome, reducing misdiagnosis and improving efficiency of resource utilization. The guideline committee proposed a consensus-based risk stratification tool to aid in pretest probability assessment for those at risk of acute aortic syndrome (RIPP score shown in Table 1).

The aim of this study is to validate the diagnostic accuracy of this tool and assess its performance in comparison to other risk prediction tools that have been developed.

## 2. Methods

This was a historical case-control study. Consecutive cases were recruited from three academic emergency departments from 2002–2020, with an annual census ranging from 75,000–120,000. Consecutive controls were recruited from a single academic emergency department. These departments were geographically distributed across Ontario, with one northern and two southern sites. This study follows the methodological and reporting recommendations outlined in the Standards for eporting of Diagnostic Accuracy Studies (STARD) criteria. [12] The study was approved by the research ethics boards of all participating institutions.

### 2.1. Data Extraction

The data were extracted and verified from multiple sources, including emergency department records and progress notes. Trained reviewers used electronic data forms and underwent training [13]. The data were analysed for agreement using the Kappa statistic with oversight. Interobserver agreement was calculated for at least 20% of the total charts. Reviewers were not informed of the study objective but were aware of the patient's case or control status.

### 2.2. Variables

The full data dictionary is included in the appendix. Abrupt onset pain was defined as pain described as sudden, unexpected, or onset at a specific time point. Severe was a pain score greater than 6 or required opioids for pain relief [14, 15]. Migrating/radiating pain was defined as pain that moved from one location to another or pain reported in two distinct anatomical locations, i.e., chest and back. Hypotension was defined by a systolic blood pressure <90 mmHg [15]. The clinical suspicion variable had three levels: AAS as most likely diagnosis, alternative diagnosis as more likely, and unsure. AAS was considered the most likely diagnosis if, after the initial assessment, a CT aorta was ordered to rule out AAS. An alternative diagnosis was considered most likely if documentation of a specific discharge or admission diagnosis was present. The clinical impression was deemed unsure if the diagnosis was unspecified or not yet diagnosed (i.e., chest pain not yet diagnosed).Where a score offered three risk levels, we dichotomized them into high risk and low risk.

### 2.3. Participants

#### 2.3.1. Case

We included consecutive eligible cases of acute aortic syndrome from 2002 to 2020. Cases were identified through an admission, discharge, or death certificated diagnosis of acute aortic syndrome.

#### 2.3.2. Control

Controls were identified through the presenting complaint of chest, abdominal, flank, back pain, and/or perfusion deficit (limb ischemia, cerebrovascular accident possible, neurological deficit, syncope, or altered level of consciousness). These were based on the Canadian Emergency Department Information System (CEDIS), which presents complaints that are indexed in our electronic health records. We randomly selected an equal number of unmatched controls (1 : 1) to be included in the final population.

#### 2.3.3. Exclusion

We excluded patients <18 years old, with a pain duration of >14 days, or with trauma within 24 hours of the onset of pain. No documented chest, abdomen, back pain, or perfusion deficit. Left without being seen or with no documentation available for the patient. A clear alternative diagnosis after initial clinical assessment, i.e., subcutaneous abscess, urinary tract infection, trapezius muscle strain, panic attack, cannabis hyperemesis syndrome, gastroenteritis, upper respiratory tract infection, uterine prolapse, upper gastrointestinal bleed, etc.

#### 2.3.4. Outcome Measures

Radiological evidence of aortic dissection, intramural hematoma, or penetrating atherosclerotic ulcer was used to identify acute aortic syndrome. The absence of acute aortic syndrome was verified through imaging. For patients who were not imaged, their medical records were reviewed for up to six months postencounter to ensure that no new diagnosis of acute aortic syndrome was made. Follow-up visits to hospitals without a diagnosis of acute aortic syndrome or imaging that did not reveal acute aortic syndrome were used to confirm the absence of the condition. In cases where patients did not return to study hospitals or undergo further imaging, publicly available sources such as obituaries were searched to determine if they had passed away.

#### 2.3.5. Data Analyses

We calculated the classification performance of the RIPP score using sensitivity and specificity together with 95% confidence intervals. We also assessed the classification of the acute aortic dissection detection risk score (ADDRS), the Lovy clinical decision tool, the Von Kodolitsch decision tool, and the AORTAs score according to their ability to classify patients as either low or high risk [16–19]. We compared the clinical decision tools' C statistic (area under the curve) and used the DeLong method to test for the significance of these differences. We calculated the absolute net reclassification indices by comparing the RIPP score with the other clinical decision tools [20]. The net reclassification index quantifies the improvement in prediction when comparing prediction tools. The sample size was based on an estimation of the precision of the classification performance of the risk scale. Our goal was to ascertain sufficient cases to evaluate the sensitivity with 95% confidence bands plus/minus 5%, corresponding to 200 cases of acute aortic syndrome.

#### 2.3.6. Patient and Public Involvement

Neither patients nor the public were formally involved in the planning of the study. We plan to involve patients before assessing the effects of implementing this rule in clinical practice.

## 3. Results

**Figure 1:**
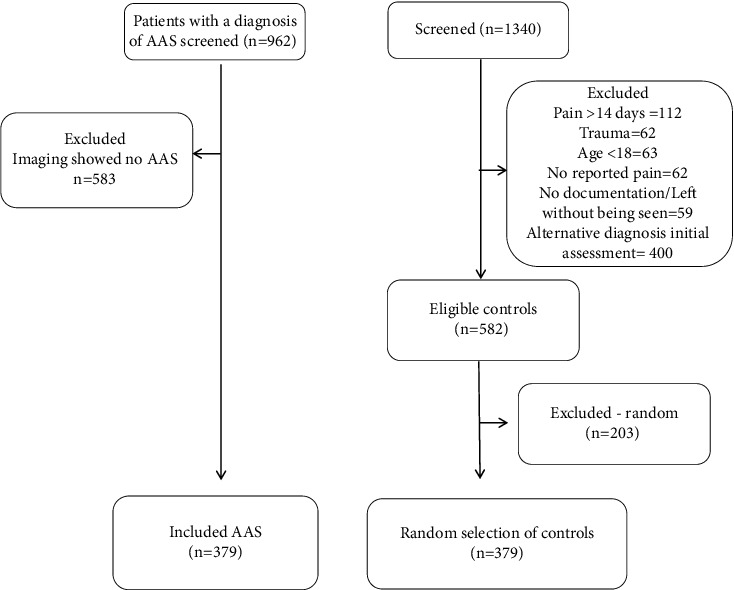
Flowchart of included patients.

Data were collected from 2002 to 2021, yielding 379 cases of acute aortic syndrome. We found 1340 potential eligible controls over a 1-month period; 580 were excluded. Of the remaining controls, 379 patients were randomly selected for the final population (Figure 1).

The Kappa between data extractors after chart training was 0.87. Chest pain unspecified (20.7%), abdominal pain unspecified (9.9%), and acute coronary syndrome (8.7%) were the top three diagnoses in the control population.  Table 2 shows the clinical features of our cohort. The patients had a mean age of 68.5 years, and 52.3% were female.

**Figure 2:**
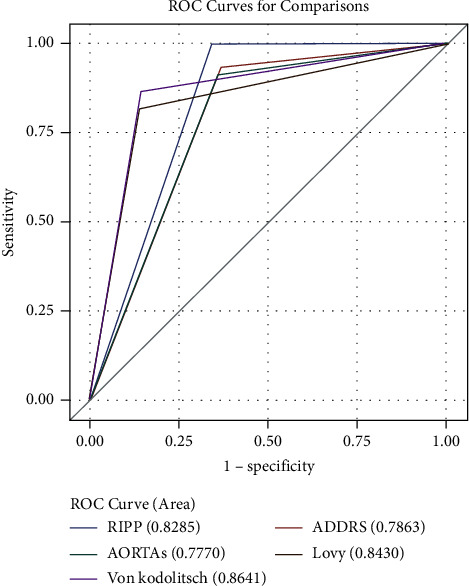
Comparison of ROC curves for each risk prediction tool.

The RIPP score had a sensitivity of 99.7%. This was higher than all the other scores. This higher sensitivity resulted in a lower specificity (53%) compared to the other clinical decision aids (63–86%) (Table 3). The DeLong comparison of the C statistics found the RIPP score had a higher C statistic compared to the ADDRS (−0.0423 (95% confidence interval −0.07–0.02); *P* < 0.0009) and the AORTAs score (−0.05 (−0.07 to −0.02); *P* = 0.0002), no difference compared to the Lovy decision tool (0.02 (95% CI −0.01–0.05 *P* < 0.25)), and decreased compared to the Von Kodolitsch decision tool (0.04 (95% CI 0.01–0.07 *P* < 0.008)) (Figure 2.  Table 4).

Table 5 shows the absolute net reclassification index between the RIPP score and the other clinical decision tools. This ranged from 0.03 compared to the AORTAs to 0.20 for the Von Kodolitsch score. The RIPP score had the highest number of cases of acute aortic syndrome that were correctly classified as high-risk.

## 4. Discussion

Our study provisionally validates the predictive performance of the RIPP score in a broad sample of patients identified in the emergency department with a diagnosis of acute aortic syndrome. To improve the generalisability of the score, we included both large and smaller volume centres, including sites that were not involved in the derivation of any of the assessed scores. The score was able to correctly stratify many more patients with acute aortic syndrome into an appropriate high-risk category. This rule meets the prespecified sensitivity identified by emergency medicine clinicians to successfully be used in guiding investigations for patients with acute aortic syndrome [21].

It should be noted that this is a retrospective case-control study and suffers from significant bias in the assessment of diagnostic accuracy estimates. This bias should affect all scores equally and therefore be nondifferential. However, this study only provides provisional evidence of diagnostic accuracy; it is not sufficient in isolation to change practice.

### 4.1. Comparison with Other Studies

We had previously surveyed emergency physicians to identify thresholds of acute aortic syndrome risk that would alter clinical decisions. In this study, respondents indicated that patients with a subsequent risk of acute aortic syndrome below 1% were most appropriate for no further investigation [21].

In our study, the RIPP score was the only decision tool that had sufficiently high sensitivity to be able to define a no testing group. The ADDRS and the AORTA scores are designed to be used with D-dimer and allow the user to differentiate between D-dimer versus computed tomography as the first test in an investigation [14, 19]. Our study confirms that they are unable to identify a low-risk group that requires no further testing, and their use should be confined to assessing D-dimer versus computed tomography as the initial investigation. It was expected that there would be a high level of missing data for D-dimer within our cohort; therefore, a priori, we decided to assess each decision aids ability to define a no testing cohort.

The Lovy and Von Kodolitsch decision aids both use clinical variables to define a low- or a high-risk group of patients. To our knowledge, this is the first evaluation of these decision aids. We found that neither had sufficient sensitivity to define a no testing group.

### 4.2. Limitations

The case-control design of this study does not allow us to define the number of selected cases and control participants relative to the source population. Therefore, baseline risks, hazards, and absolute probabilities cannot be correctly adjusted. However, the risk of bias is applicable to all decision tools tested, and the comparative differences are not affected.

One variable of the score was clinical impression. This was extracted from the patient chart; therefore, a physician may have had a clinical suspicion for AAS but not documented the diagnosis. This may artificially inflate the specificity of the clinical risk score. We were conservative in our estimate of the presence of a clinical impression for AAS, only coding as yes if a CT aorta was ordered to rule out AAS; therefore, the inherent fragility of this variable should not affect the reported sensitivity.

Our aim was to select a control population that reflected those in whom there would be a reasonable consideration of acute aortic syndrome. We excluded those who had an image-proven alternative diagnosis or those who underwent computed tomography with intravenous contrast for another reason and would have been diagnosed with acute aortic syndrome. Because of this, we were left with a population that was less severe in presentation than those who underwent imaging for other reasons. This likely artificially inflates our specificity; however, the comparative differences in specificity between tools should be independent of this bias.

This was a retrospective chart review where missing values were defaulted to negative. This was done to bias our results towards the lowest possible sensitivity. In addition, no assessment of the interrater reliability of the clinical variables could be performed.

Our cases are patients in whom acute aortic syndrome was identified; patients who were never identified, discharged, or died will not be identified. These patients are likely to have a different presentation, and thus the RIPP score may not perform as well in this patient population.

Not all patients underwent the reference standard of computed tomography, MRI, or transesophageal echocardiography. Therefore, there is a possibility of misclassifying cases as controls. This could artificially increase our sensitivity and specificity. We could potentially have chosen a control population from patients who underwent imaging to rule out acute aortic syndrome; however, that would have resulted in partial verification bias and artificially lowered the specificity.

### 4.3. Research and Clinical Implications

A prospective multicentre implementation study following established implementation guidelines is now needed to assess the impact of the RIPP score when applied in clinical practice. Although a tool may have sufficient accuracy to classify cases and controls, it still may not lead to the desired decrease in missed cases of AAS or the efficient use of advanced imaging.

The major concern with all of the clinical risk scores is the likely low specificity in clinical practice [22]. We found that the RIPP score had the lowest specificity. In order for the RIPP score to achieve sufficient sensitivity to meet prespecified criteria, there is a corresponding increase in false positives. The question remains: is the potential decrease in the missed rate of this rare diagnosis adequately balanced with the likely increase in CT usage? Although clinicians may use the score to help guide them in the collection of important clinical variables to help define risk for AAS, caution should be used until an implementation study is completed in order to confirm its use leads to better patient outcomes. Clinical decision rules and risk scores, when implemented, may not lead to a clinically important change in patient-oriented outcomes [22].

## 5. Conclusion

The RIPP score identifies those who require further investigation for acute aortic syndrome among those presenting to the emergency department with undifferentiated symptoms. Incorporating this validated clinical decision tool into clinical practice has the potential to improve decisions regarding the investigation of patients for acute aortic syndrome in the emergency department.

## Figures and Tables

**Table 1:** RIPP score. A consensus-based clinical decision aid to help guide investigation for acute aortic syndrome based on risk factors, impression, physical exam, and description of pain. Score <1 means no further investigation, and >1 means further investigation required.

RIPP score		
Risk factor	No risk factor	0 points
	Nonaneurysmal risk factor	1 points
	Aortic aneurysm	2 points

Impression	Alternative diagnosis likely	−1 points
	Unsure	0 points
	AAS most likely	2 points

Physical exam	No physical exam findings	0 points
	Any physical exam findings	2 points

Pain	No high risk pain features	0 points
	1 or 2 high risk pain features	1 points
	>2 high risk pain features	2 points

**Table 2:** Characteristics of patients with AAS (acute aortic syndrome) and controls.

Characteristics	*N* (%)	
Risk factors	AAS (*N* = 379)	Controls (*N* = 379)
Family history	6 (1.5)	1 (0.2)
Aortic manipulation	1 (0.3)	0
Aortic valve disease	14 (3.7)	0
Known aortic aneurysm	69 (18.2)	2 (0.5)
Known thoracic aortic aneurysm	23 (6)	0
Connective tissue disease	1 (0.2)	0
Pain		
Abrupt onset pain	308 (81.2)	50 (13.2)
Tearing/ripping	22 (5.8)	1 (0.26)
Migrating/radiating pain	302 (79.7)	62 (16.4)
Severe	121 (24.6)	117 (23.9)
Physical exam		
Hypotension	53 (13.9)	1 (0.3)
New murmur	16 (4.2)	0
Pulse deficit	20 (5.3)	1 (0.3)
Focal neurological deficit	41 (10.8)	0
Bilateral blood pressure differential >20 mmHg	29 (7.6)	2 (0.5)
Impression		
Clinical suspicion for an alternative diagnosis	40 (10.6)	113 (29.8)
Investigations		
CXR abnormal	51 (13.1)	0

**Table 3:** Diagnostic accuracy of risk prediction tools for acute aortic syndrome.

	TP	FN	TN	FP	Sensitivity	Specificity	AUC	Pr > Chisq
ADDRS	355	24	241	138	93.67 (90.72–95.90)	63.59 (58.52–68.44)	0.7861	0.0009
LOVY	310	69	329	50	81.79 (77.53–85.55)	86.81 (82.98–90.05)	0.8468	0.36
AORTAs	346	33	243	136	91.29 (87.99–93.93)	64.12 (59.06–68.95)	0.77	0.0002
Von Kodolitsch	329	50	326	53	86.81 (82.98–90.05)	86.02 (82.11–89.35)	0.8691	0.02
RIPP	378	1	201	178	99.74 (98.54–99.99)	53.03 (47.87–58.15)	0.8284	—

TP: true positive; FN: false negative; TN: true negative; FP: false positive; ADDRS: acute aortic dissection detection risk score.

**Table 4:** Area under the curve for each prediction tool. The difference in area under the curve for each tool in comparison to the RIPP score. The RIPP score has a higher AUC than the ADDRS and the AORTAs, and a lower AUC than the Von Kodolitsch score and Lovy decision aid.

	Area under the curve (AUC)	AUC difference	Pr > Chisq
ADDRS	0.79	−0.04	0.0009
LOVY	0.84	0.02	0.36
AORTAs	0.77	−0.05	0.0002
Von Kodolitsch	0.87	0.04	0.02
RIPP	0.83	—	—

**Table 5:** Net reclassification index for risk prediction tools in comparison to the RIPP score. RIPP score performed better than all comparator scores in classifying cases of acute aortic syndrome, improving reclassification (range 11–34%). All comparator scores performed better than the RIPP score decreasing misclassification of those without acute aortic syndrome (6–18%).

	Net reclassification index	Low to high	High to low	*P* value
ADDRS	0.05	−0.06	0.11	*P* < 0.002
LOVY	0.16	−0.18	0.34	*P* < 0.0001
AORTAs	0.03	−0.08	0.11	*P* < 0.002
Von Kodolitsch	0.20	−0.13	0.33	*P* < 0.0001
RIPP	—	—	—	—

## Data Availability

The data used to support the findings of this study are available from the corresponding author upon reasonable request.

## Supplementary Materials

Supplementary Materials Appendix-Data dictionary with concise definitions used for data extraction.
