# Supplementary material for: Evaluation of the Canadian Clinical Practice Guidelines Risk Prediction Tool for Acute Aortic Syndrome: The RIPP Score
Source: Emerg Med Int. 2023 May 25;2023:6636800. doi: 10.1155/2023/6636800 (PMC10234704; doi:10.1155/2023/6636800)
Supplement: Supplementary Materials — Appendix-Data dictionary with concise definitions used for data extraction. [file 6636800.f1.docx]

Appendix

**Consensus Definitions – Aortic Dissection Study**

Patient Identifiers

- Patient identifiers were recorded from the patient identification section of each chart generated in triage.
- **Subject Number:** Each participant was randomly assigned a unique identifier.
- **HSN Number:** Each participant’s unique Health Sciences North (HSN) identifier was recorded.
- **OHIP Number:** Each participant’s unique OHIP identifier was recorded.
- **Name:** The patient’s first, last, and any middle names were recorded.
- **Date of Birth:** The patient’s date of birth was recorded in a day/month/year (dd/mm/yyyy) format.
- **Age:** The patient’s age was recorded in years.
- **Sex**: The patient’s sex was recorded as either male or female.
- **Visit Date:** Each patient’s visit date to Health Sciences North was recorded (dd/mm/yyyy).

Mode of Arrival

- **Referred by Other Healthcare Provider:** Patient was coded as a “yes” if the chart explicitly stated that they were referred to the ED by another healthcare provider or were transferred to HSN after being seen at a peripheral ED. E.g. A Triage Note saying, “Pt was seen as at walk in clinic and told to come to ED”. Otherwise, the patient was assumed to not have been referred to the ED and was coded as a “no”.

Triage Chart Used

- Each patient presenting to HSN was assigned a specific chart type corresponding to their chief complaint at triage. The patient was coded as a “yes” based on the type of chart they received (below). Patients are coded as “Other” for all other chart types.
  - **Chest pain – cardiac possible**
  - **Chest pain – non cardiac**
  - **Coma / altered consciousness**
  - **Abdominal pain**
  - **Flank pain**
  - **Back pain**
  - **Extremity complaint**
  - **Syncope / loss of consciousness**
  - **Stroke / TIA**
  - **Hypertension**
  - **Hypotension**
  - **Other**

Exclusions

- **Pain > 14 days:** Patient was coded as a “yes” if the chart explicitly stated that the same character of pain was present for a duration greater than 14 days prior to the emergency department visit. If the onset of pain was explicitly recorded in the chart as starting less than 14 days prior to the emergency department visit or the specific time of onset was not recorded at all, the patient was coded as a “no.”
- **Due to Trauma:** Patient was coded as a “yes” if chart explicitly correlated a traumatic event to the symptom onset that brought the patient to the emergency department. If the traumatic event was seemingly minor (such as a fall from standing height), a “yes” was coded and the mechanism was recorded in the “Other Notes” section. If no trauma occurred, or no mention of trauma was found in the chart, the patient was coded as a “no.”
- **<18 Years of Age:** Patient was coded as a “yes” if they were under 18 years of age.
- **Missing chart:** Patient was coded as a “yes” if no emergency department chart was available for review.
- **Incidental Finding:** Patient was coded as a “yes” if an aortic dissection was incidentally found on imaging and the asymptomatic patient was then asked to present to the emergency department.

Symptom

- **Abrupt-Onset of Pain**: Patient was coded as a “yes” if the pain onset was documented as: acute, sudden or at a defined time. If the onset was gradual, the patient was coded as a “no.” If the chart did not explicitly indicate the nature of pain onset, then the data point was left blank.
- **Maximal Intensity at Onset**: Patient was coded as a “yes” if the pain was maximal in intensity at its advent or peaked within 1 minute. Pain described as “thunderclap” also met this criteria. If the pain became worse after the onset - within a 6hr window - the patient was coded as a “no.” If the chart did not explicitly indicate the nature of the pain at the onset, then the data point was left blank.
- **Severe Pain**: Patient was coded as a “yes” if the pain was described as severe. Pain described as “worst pain ever” or ≥7/10 also met this criteria. If the pain was not described as severe, the patient was coded as a “no.” If the chart did not explicitly indicate the severity of the pain, then the data point was left blank.
- **Pain Ongoing**: Patient was coded as a “yes” if the pain was ongoing at the time of the patient-physician encounter on the basis of either the history or physical exam. If the pain had resolved at this juncture, the patient was coded as a “no.” If the chart did not explicitly indicate if the patient was experiencing pain when the physician examined them, then the data point was left blank.
- **Location of Pain**: The primary location of pain was coded based on anatomic area (below) as from history or physical exam. If more than one location of pain was present and not deemed to be radiating pain as per the physician, all of the corresponding affected areas were coded as a “yes.” The patient was coded as a “no” if the chart explicitly indicated that the patient was not having pain in a specific anatomic area or it was an area that primary pain was radiating to. If the chart did not explicitly comment on the anatomic area, then the data point was left blank.
  - **Back**
  - **Abdomen**
  - **Chest**
  - **Flank**
  - **Arm**
  - **Leg**
- **Character of Pain**: Patient was coded as a “yes” if their pain was described using one of the descriptors below. If more than one descriptor of pain was recorded, all of the corresponding descriptors were coded as a “yes.” The patient was coded as a “no” if the chart explicitly indicated that the patient was not having a specific character of pain. If the chart did not explicitly comment on the character of pain, then the data point was left blank.
  - **Tearing/ripping**
  - **Sharp**
  - **Squeeze/pressure/tight**
  - **Pleuritic**
  - **Other (examples: burning, cramping or indescribable)**
- **Migrating/Radiating Pain**: The patient was coded as a “yes” if pain migrated/radiated from the primary anatomic area to the same or different anatomic area (below) as from history or physical exam. If the pain migrated/radiated to more than one anatomic location, all of the corresponding affected areas were coded as a “yes.” The patient was coded as a “no” if the chart explicitly indicated that the patient was not having migrating/radiating pain. If the chart did not explicitly comment on if the patient was having migrating/radiating pain then the data point was left blank.
  - **Neck**
  - **Arm**
  - **Leg**
  - **Back**
  - **Flank**
  - **Abdomen**
  - **Chest**
- **Nausea or vomiting:** Patient was coded as a “yes” if the chart explicitly states that the patient had nausea or vomiting in the pre-hospital setting or emergency department. Patient was coded as a “no” if the chart explicitly states that the patient did not have nausea or vomiting. If the chart does not explicitly comment on if the patient had nausea/vomiting, then the data point was left blank.
- **Diaphoresis:** Patient was coded as a “yes” if the chart explicitly states that the patient had diaphoresis in the pre-hospital setting or emergency department. Patient was coded as a “no” if the chart explicitly states that the patient did not have diaphoresis. If the chart does not explicitly comment on if the patient had diaphoresis, then the data point was left blank.
- **Syncope/loss of consciousness**:Patient was coded as a “yes” if the chart explicitly states that the patient had a syncopal event or lost consciousness either in the pre-hospital setting or emergency department. Patient was coded as a “no” if the chart explicitly states that the patient did not have a syncopal event or lost consciousness. If the chart does not explicitly comment on if the patient had a syncopal event or lost consciousness, then the data point was left blank.
- **Presyncope**:Patient was coded as a “yes” if the chart explicitly states that the patient had a presyncopal event either in the pre-hospital setting or emergency department. Other synonymous terms for presyncope include “near loss of consciousness.” Patient was coded as a “no” if the chart explicitly states that the patient did not have a presyncopal event. If the chart does not explicitly comment on if the patient had a presyncopal event, then the data point was left blank.
- **Subjective Neurological Deficits**: Patient was coded as a “yes” if the patient described focal weakness or altered sensation, resolved or ongoing. Patient was coded as a “no” if the chart explicitly states that the patient did not have these subjective neurological deficits. If the chart does not explicitly comment on if the patient had a subjective neurological deficits, then the data point was left blank.

PMH

- **Recent Aortic Manipulation:** Patient was coded as “yes” if the chart explicitly states anywhere that the patient had a recent aortic manipulation as defined by surgery involving either the thoracic or abdominal aorta within the past 4 weeks. The patient was coded “no” if the chart indicates explicitly that the patient did not have aortic manipulation within the past 4 weeks or there is no past medical history. If the above criteria are not explicitly indicated in the chart then this data point was left blank.
- **Aortic Valve Disease:** Patient was coded as “yes” if the chart explicitly states anywhere that the patient has aortic valve disease defined by either a bicuspid aortic valve or the patient having surgical/endovascular repair/graft replacement for previous aortic valve disease. The patient was coded “no” if the chart indicates explicitly that the patient does not have aortic valve disease or there is no past medical history. If the above criteria are not explicitly indicated in the chart then this data point was left blank. If the patient had aortic valve disease, the type was recorded below:
  - **Bicuspid Valve**
  - **Aortic Valve Replacement**
- **Aortic Stenosis or Insufficiency:** Patient was coded as “yes” if the chart explicitly states anywhere that the patient has aortic valve disease defined by either aortic stenosis or aortic insufficiency. The patient was coded “no” if the chart indicates explicitly that the patient does not have aortic stenosis or aortic insufficiency or there is no past medical history. If the above criteria are not explicitly indicated in the chart then this data point was left blank
- **Known Abdominal Aortic Aneurysm:** Patient was coded as “yes” if the chart explicitly states anywhere that the patient has a known abdominal aortic aneurysm prior to formal imaging ordered by the emergency department physician. The patient was coded “no” if the chart indicates explicitly that the patient does not have a known abdominal aortic aneurysm or there is no past medical history. If the above criteria are not explicitly indicated in the chart then this data point was left blank.
  - **Size of Abdominal Aortic Aneurysm:** If an aneurysm was known, its size was documented verbatim.
  - **AAA discovered on ED POCUS:** Patient was coded as “yes” if the chart explicitly states anywhere that the patient’s AAA was first discovered by the emergency department physician on POCUS. The patient was coded “no” if the chart indicates explicitly that the POCUS was not performed or POCUS was performed and an AAA was not found. If the above criteria are not explicitly indicated in the chart then this data point was left blank.
- **Known Thoracic Aortic Aneurysm:** Patient was coded as “yes” if the chart explicitly states anywhere that the patient has a known thoracic aortic aneurysm prior to formal imaging ordered by the emergency department physician. The patient was coded “no” if the chart indicates explicitly that the patient does not have a known thoracic aortic aneurysm or there is no past medical history. If the above criteria are not explicitly indicated in the chart then this data point was left blank.
  - **Size or Thoracic Aortic Aneurysm:** If an aneurysm was known, its size was documented verbatim.
- **AAA Repair:** Patient was coded as “yes” if the chart explicitly states anywhere that the patient had a prior AAA repair. The patient was coded “no” if the chart indicates explicitly that the patient has not had a prior AAA repair or there is no past medical history. If the above criteria are not explicitly indicated in the chart then this data point was left blank.
- **Ischemic Heart Disease:** Patient was coded as “yes” if the chart explicitly mentions the patient has had a myocardial infarction, heart attack, coronary artery disease, angina, or CABG operation. The patient was coded “no” if the chart explicitly indicates that none of the above heart conditions were present or there is no past medical history. If the chart did not explicitly indicate a presence or absence of an ischemic heart disease history then the data point was left blank.
- **Connective Tissue Disease:** Patient was coded as “yes” if the chart explicitly states that the patient has a history of a connective tissue disease such as Marfan’s Syndrome or Ehlers-Danlos Syndrome. The patient was coded “no” if the chart indicates explicitly that the patient has no connective tissue disease history/features or there is no past medical history. If the chart did not explicitly indicate a presence or absence of a connective tissue disease then the data point was left blank.
- **Hypertension**: Patient was coded as “yes” if the chart explicitly states that the patient has a history of hypertension or if the patient is regularly takes an anti-hypertensive medication. The patient was coded “no” if the chart indicates explicitly that the patient does not have hypertension or there is no past medical history. If the chart did not explicitly indicate a presence or absence of the above, then the data point was left blank. Note: hypertensive blood pressure(s) recorded in the pre-hospital setting or emergency department did not classify the patient as having underlying hypertension.
- **Diabetes:** Patient was coded as “yes” if the chart explicitly states anywhere that the patient has diabetes or if the patient is taking a diabetes medication such as insulin, metformin, etc. The patient was coded “no” if the chart indicates explicitly that the patient does not have diabetes or there is no past medical history. If the above criteria are not explicitly indicated in the chart then this data point was left blank.
- **Smoking:** Patient was coded as “yes” if the chart explicitly states anywhere that the patient actively smokes or quit smoking within the past 6 months. The patient was coded “no” if the chart indicates explicitly that the patient does not smoke or quit greater than 6 months ago. If the above criteria are not explicitly indicated in the chart then this data point was left blank.
- **Family History of Acute Aortic Syndrome:** Patient was coded as “yes” if the chart explicitly states anywhere that the patient has a first degree relative with history of acute aortic syndrome. The patient was coded “no” if the chart indicates explicitly that the patient does not have a family history of acute aortic syndrome. If the above criteria are not explicitly indicated in the chart then this data point was left blank.
- **Family History of Abdominal Aortic Aneurysm:** Patient was coded as “yes” if the chart explicitly states anywhere that the patient has a first degree relative with history of abdominal aortic aneurysm. The patient was coded “no” if the chart indicates explicitly that the patient does not have a family history of abdominal aortic aneurysm. If the above criteria are not explicitly indicated in the chart then this data point was left blank.
- **Known Aortic Dissection:** Patient was coded as “yes” if the chart explicitly states anywhere that the patient has a known aortic dissection prior to formal imaging ordered by the emergency department physician. The patient was coded “no” if the chart indicates explicitly that the patient does not have a known abdominal aortic aneurysm or there is no past medical history. If the above criteria are not explicitly indicated in the chart then this data point was left blank.
- **Aortic Dissection Repair:** Patient was coded as “yes” if the chart explicitly states anywhere that the patient has had a prior aortic dissection that was surgically repaired. The patient was coded “no” if the chart indicates explicitly that the patient has never had an aortic dissection that was surgically repaired or there is no past medical history. If the above criteria are not explicitly indicated in the chart then this data point was left blank.

Physical Exam

- **GCS Score:** The GCS (scale 3-15) on the emergency department chart was recorded verbatim. If the chart did not explicitly indicate the GCS, then the data point was left blank. If more than one GSC score was recorded, the first documented GCS was used.
- **Bilateral BP measurement**: Measured in arms within 12 hours of presentation. If more than one measurement, the first documented BP was recorded. Patient was coded as “yes” if systolic BPs in bilateral arms are >20mmHg and “no” if ≤20. If bilateral blood pressures are not explicitly recorded in the chart then this data point was left blank.
- **Triage systolic BP measurement**: Systolic blood pressure recorded at triage was recorded verbatim. If a triage blood pressure was not explicitly recorded in the chart then this data point was left blank.
- **Triage diastolic BP measurement**: Diastolic blood pressure recorded at triage was recorded verbatim. If a triage blood pressure was not explicitly recorded in the chart then this data point was left blank.
- **Hypertension** (>150mmHg): the earliest document blood pressure (most often the BP taken at triage; not in the pre-hospital setting). Patient was coded as “yes” if systolic BP is >150mmHg and “no” if ≤150. If blood pressure is not explicitly recorded in the chart then this data point was left blank.
- **Hypotension** (<90mmHg): any documented hypotension within the emergency department (not in the pre-hospital setting). Patient was coded as “yes” if systolic BP is <90mmHg and “no” if ≥90. If blood pressure is not explicitly recorded in the chart then this data point was left blank.
- **New murmur**: Patient was coded as “yes” for any new murmur heard on auscultation (not specified if diastolic or systolic). If the murmur is not known to be new or old, it is defined as new. If heart sounds are auscultated, but a heart murmur is not explicitly recorded in the chart then this data point was coded as a “no”. If heart sounds are not explicitly recorded in the chart then this data point was left blank.
- **Pulse deficit**: Patient was coded as “yes” if there is asymmetry in character of pulse or new asymmetrical signs of an ischemic limb. Patient was coded as “no” if the chart explicitly indicates that the patient does not have a pulse deficit. If the chart does not explicitly indicate the presence or absence of a pulse deficit then the data point was left blank.
- **Other Objective Sensory Neurological Signs:** Patient was coded as “yes” if the chart explicitly indicates some objective sensory neurological deficit on physical exam. Patient was coded as “no” if the chart explicitly indicates that there were no objective sensory neurological deficits on physical exam. If the chart does not explicitly indicate the presence or absence of sensory neurological deficits then the data point was left blank.
- **Other Objective Motor Neurological Signs:** Patient coded as “yes” if the chart explicitly indicates some objective motor neurological deficit such as weakness, absent reflexes, or a cranial nerve deficit on physical exam. Patient coded “no” if the chart explicitly indicates that there were no objective motor function deficits on physical exam. If the chart did not explicitly indicate a presence or absence of objective motor function deficits then the data point was left blank.

Arrest

- **Patient arrested:** Patient coded as a “yes” if the chart indicates that the patient required chest compressions while in the ED. Patient coded as a “no” if the chart does not indicate that an arrest occurred.
- **ROSC:** Patient coded as a “yes” if the chart indicates that the patient required chest compressions and had sustained ROSC allowing for transfer from the ED to a consulting service. Patient coded as a “no” if the chart indicates that the patient required chest compressions and died while in the ED. If the chart does not indicate that the patient arrested, then the data point was left blank.

Blood Work

- **Blood work:** Patient was coded as a “yes” if blood work was performed in the emergency department. Patient was coded as a “no” if blood work was not performed in the emergency department. Blood work results are analysed below:
  - **Hemoglobin (in ER):** The hemoglobin value (g/L) was recorded verbatim. If an emergency department hemoglobin was not drawn, then the data point was left blank.
  - **D-dimer >500ng/dl:** The patient was coded as a “yes” if a d-dimer was drawn and the result was > 500ng/dl. The patient was coded as a “no” if the d-dimer was ≤ 500ng/dl. If a d-dimer was not drawn, then the data point was left blank.
    - **D-dimer value:** If a d-dimer was drawn, the result (ng/dl) was recorded verbatim.
  - **Troponin I Elevated (in ER):** The patient was coded as a “yes” if a troponin I was drawn and any result while in the emergency department was elevated as per Health Sciences North laboratory normals (> 0.012g/L). The patient was coded as a “no” if the troponin I was normal (≤ 0.012g/L). If a troponin was not drawn, then the data point was left blank.
  - **Troponin I delta > 25% (in ER):** The patient was coded as a “yes” if troponin I was quantified in serial blood work (using the same assay type) while in the emergency department and there is > 25% change between two troponin readings. The patient was coded as a “no” if delta troponin I changes are ≤ 25%. If serial troponins are not drawn, then the data point was left blank.

Imaging

- Imaging is only relevant if it was performed at the ER visit, or was associated with the original ER presentation (for example, imaging ordered by the ER physician but completed after patient admission). Incidental findings were not recorded below.
- **CT performed:** binary “yes” or “no.” If “yes,” the anatomic area for which the scan is requested was coded as a “yes” (below). If a CT scan is directed to more than one anatomic area, a “yes” was coded for each anatomic area. A “no” was coded for all anatomic areas the CT is not explicitly ordered for.
  - **CT Thorax**
  - **CT Abdomen**
  - **CT Aorta**
  - **CT Lower Extremities**
- **Indication for CT:** If CT was performed, the indication for CT was recorded verbatim. If the chart did not explicitly indicate the indication for CT, then the data point was left blank.
- **CT abnormal findings:** If CT was performed, the patient was coded as a “yes” if there were abnormal findings on CT. The patient was coded as a “no” if there are no abnormal findings on CT. If there are abnormal findings, these abnormal findings were recorded verbatim.
- **Time CT ordered:** If CT/CTA was performed, the time the CT/CTA was ordered (whichever was ordered first) by the emergency department physician was recorded verbatim. If the time was not recorded, this data point was left blank.
- **Time CT performed:** If CT/CTA was performed, the time the CT/CTA was performed (whichever was performed first) was recorded verbatim. If the time was not recorded, this data point was left blank.
- **CTA performed:** binary “yes” or “no.” Patient was coded as “no” if a CTA was not explicitly performed as per the chart.
- **Indication for CTA:** If CTA was performed, the indication for CTA was recorded verbatim. If the chart did not explicitly indicate the indication for CT, then the data point was left blank.
- **CTA abnormal findings:** If CTA was performed, the patient was coded as a “yes” if there are abnormal findings on CTA. The patient was coded as a “no” if there are no abnormal findings on CTA. If there are abnormal findings, these abnormal findings were recorded verbatim.
- **Coronary angiogram performed:** binary “yes” or “no.” Patient was coded as “no” if a coronary angiogram was not explicitly performed as per the chart.
- **Coronary Angiogram abnormal findings:** If coronary angiogram was performed, the patient was coded as a “yes” if there were abnormal findings on CTA. The patient was coded as a “no” if there were no abnormal findings on CTA.
- **Chest X-ray performed:** binary “yes” or “no.” Patient was coded as “no” if a chest x-ray was not explicitly performed as per the chart.
- **Chest X-ray abnormal findings:** If chest x-ray was performed, the patient was coded as“yes” if there were abnormal findings of widened mediastinum or absence of aortic notch explicitly stated on the chart. The patient was coded as “no” if these abnormal findings were not explicitly stated on the chart.
- **Bedside Echo performed:** binary “yes” or “no.” Patient was coded as “yes” if a bedside echo (either TTE or TEE) was explicitly performed by an emergency department physician or another experienced provider as per the chart. Patient is coded as “no” if a bedside echo was not explicitly performed as per the chart.
- **Bedside Echo abnormal findings:** If bedside echo was performed, the patient was coded as “yes” if there were abnormal findings of valve abnormalities explicitly stated on the chart. The patient was coded as “no” if these abnormal findings were not explicitly stated on the chart.
- **Time at imaging:** The time that confirmatory imaging of an aortic dissection was performed (not as formally dictated by Radiology).

Aortic Dissection Type/Features

- If an aortic dissection is confirmed through advanced imaging (CT, CTA, MRI or TEE), its features are recorded below:
- **Chronic event:** Patient was coded as a “yes” if aortic pathology on imaging is believed to represent a chronic aortic dissection. Patient was coded as a “no” if the chronicity of the aortic dissection is not commented on or it is felt to be acute.
- **Aortic dissection:** Patient was coded as a “yes” if aortic pathology on imaging is reported as an aortic dissection. Patient was coded as a “no” if an aortic dissection is not commented upon.
- **Intramural hematoma:** Patient was coded as a “yes” if aortic pathology on imaging is reported as an intramural hematoma. Patient was coded as a “no” if an intramural hematoma is not commented upon.
- **Penetrating ulcer:** Patient was coded as a “yes” if aortic pathology on imaging is reported as an aortic ulcer leading to an intimal tear. Patient was coded as a “no” if an aortic ulcer is not commented upon.
- **Type A:** Patient was coded as a “yes” if aortic pathology is located in the ascending aorta. Patient was coded as a “no” if aortic pathology is not located in the ascending aorta.
- **Type B:** Patient was coded as a “yes” if aortic pathology is located in the descending aorta and not the ascending aorta. Patient was coded as a “no” if aortic pathology is not located in the descending aorta or if aortic pathology is not located in both the ascending and descending aorta (Type A).
- **Proximal dissection:** The site of the most proximal extension of the dissection flap or hematoma was recorded verbatim as per the chart. If the anatomic location is not explicitly indicated in the chart, then this data point was left blank.
- **Distal dissection:** The site of the most distal extension of the dissection flap or hematoma was recorded verbatim as per the chart. If the anatomic location is not explicitly indicated in the chart, then this data point was left blank.
- **Intimal tear:** The site of the intimal tear was recorded verbatim as per the chart. If the anatomic location is not explicitly indicated in the chart, then this data point was left blank.
- **Associated vessel involvement:** Patient was coded as a “yes” if the dissection flap or hematoma compromises the patency of either proximal or distal vessels as below. Patient was coded as a “no” if the chart explicitly indicates a vessel was not involved. If the above criteria are not explicitly indicated in the chart (for example, due to uncertainty in image interpretation or they are not commented upon) then this data point was left blank.
  - **Coronary**
    - **Right**
    - **Left**
  - **Brachiocephalic**
  - **Carotid**
    - **Right**
    - **Left**
  - **Subclavian**
    - **Right**
    - **Left**
  - **Abdomen**
    - **Celiac**
    - **SMA**
    - **Renal**
    - **IMA**
    - **Iliac (at bifurcation)**
- **Pericardial effusion:** Patient was coded as a “yes” if imaging confirms the presence of a pericardial effusion. The patient was coded as a “no” if the chart explicitly indicates that the patient does not have a pericardial effusion. If the above criteria are not explicitly indicated in the chart then this data point was left blank.

Outcome

- **Admitted to Hospital:** Patient coded “yes” if the chart indicates that the patient was admitted to the hospital. Patient coded “no” if the chart indicates that the patient was discharged from the ED.
- **Discharged:** Patient coded “yes” if the chart indicates that the patient was discharged from the ED. Patient coded “no” if the chart indicates that the patient was admitted to the hospital.
- **Died within 14 days:** Patient coded “no” if hospital records indicated that they received care >14 days after initial presentation. Patient codes “yes” if hospital records or ICIS indicates that the patient died within 14 days of initial presentation. If there are no hospital records indicating care >14 days after initial presentation and no indication they died within 14 days then the data point was left blank.
  - **Reason for Death:** If the patient died within 14 days, the reason for their death was recorded verbatim if available from the chart
- **Returned to ED <14 days with any complaint:** Patient coded “yes” if hospital records indicate the patient was seen in the ED with any complaint within 14 days of initial presentation indicating a potential missed acute aortic syndrome. Patient coded “no” if hospital records indicate the patient was not seen in the ED within 14 days of the initial presentation. If “yes,” the chief complaint of the initial presentation was recorded as below:
  - **Chest pain – cardiac possible**
  - **Chest pain – non cardiac**
  - **Coma / altered consciousness**
  - **Abdominal pain**
  - **Flank pain**
  - **Back pain**
  - **Extremity complaint**
  - **Syncope / loss of consciousness**
  - **Stroke / TIA**
  - **Hypertension**
  - **Hypotension**
  - **Other**

Diagnosis

- **ED Diagnosis:** Recorded as indicated by the ED physician in the chart verbatim.
- **Hospital Discharge Diagnosis:** Recorded as indicated in the discharge summary for those patients who were admitted to the hospital verbatim. This data point was left blank in patients who were not admitted to the hospital.
- **Time at Initial Assessment:** The time of initial assessment was included whenever possible as extracted from the chart. It was recorded in dd/mm/yyyy and 24 hour time. If no time was available, this data point was left blank.
- **Time at Transfer:** If the patient was transferred from another hospital to HSN, the time of transfer was included whenever possible as extracted from the chart. It was recorded in dd/mm/yyyy and 24 hour time. If no time was available, this data point was left blank
- **Time at Discharge/Decision to Admit:** The time at discharge/decision to admit was included whenever possible as extracted from the chart. It was recorded in dd/mm/yyyy and 24 hour time. If no time was available, this data point was left blank.
- **Time in the Emergency Department:** Calculated by subtracting the time at discharge/decision to admit from the time of initial assessment. Recorded in elapsed time in hours and minutes. If no time was available for the time at initial assessment and/or time at discharge/decision to admit, this data point was left blank.

Other Notes

- **Physician name:** The name of the emergency department physician who completed (and signed off on) the chart was recorded.
- **Notes of Interest:** A space was provided on the data extraction sheet for individual reviewers to include comments they thought might be relevant.
